# A Global Overview of the Genetic and Functional Diversity in the *Helicobacter pylori cag* Pathogenicity Island

**DOI:** 10.1371/journal.pgen.1001069

**Published:** 2010-08-19

**Authors:** Patrick Olbermann, Christine Josenhans, Yoshan Moodley, Markus Uhr, Christiana Stamer, Marc Vauterin, Sebastian Suerbaum, Mark Achtman, Bodo Linz

**Affiliations:** 1 Institute for Medical Microbiology and Hospital Epidemiology, Medizinische Hochschule Hannover, Hannover, Germany; 2 Department of Molecular Biology, Max Planck Institute for Infection Biology, Berlin, Germany; 3 Applied Maths, Sint-Martens-Latem, Belgium; 4 Environmental Research Institute, University College Cork, Cork, Ireland; 5 Department of Biochemistry and Molecular Biology, Pennsylvania State University, University Park, Pennsylvania, United States of America; Fred Hutchinson Cancer Research Center, United States of America

## Abstract

The *Helicobacter pylori cag* pathogenicity island (*cag*PAI) encodes a type IV secretion system. Humans infected with *cag*PAI–carrying *H. pylori* are at increased risk for sequelae such as gastric cancer. Housekeeping genes in *H. pylori* show considerable genetic diversity; but the diversity of virulence factors such as the *cag*PAI, which transports the bacterial oncogene CagA into host cells, has not been systematically investigated. Here we compared the complete *cag*PAI sequences for 38 representative isolates from all known *H. pylori* biogeographic populations. Their gene content and gene order were highly conserved. The phylogeny of most *cag*PAI genes was similar to that of housekeeping genes, indicating that the *cag*PAI was probably acquired only once by *H. pylori*, and its genetic diversity reflects the isolation by distance that has shaped this bacterial species since modern humans migrated out of Africa. Most isolates induced IL-8 release in gastric epithelial cells, indicating that the function of the Cag secretion system has been conserved despite some genetic rearrangements. More than one third of *cag*PAI genes, in particular those encoding cell-surface exposed proteins, showed signatures of diversifying (Darwinian) selection at more than 5% of codons. Several unknown gene products predicted to be under Darwinian selection are also likely to be secreted proteins (e.g. HP0522, HP0535). One of these, HP0535, is predicted to code for either a new secreted candidate effector protein or a protein which interacts with CagA because it contains two genetic lineages, similar to *cagA*. Our study provides a resource that can guide future research on the biological roles and host interactions of *cag*PAI proteins, including several whose function is still unknown.

## Author Summary

Most humans are infected with *Helicobacter pylori*. The *H. pylori cag* pathogenicity island (*cag*PAI) encodes a secretion apparatus that can translocate the CagA protein into host cells. Humans infected with *cag*PAI–carrying *H. pylori* are at increased risk of severe disease, including gastric cancer. We analyzed the nucleotide sequences and functional diversity of the *cag*PAI in a globally representative collection of isolates. Complete *cag*PAI sequences were obtained for 29 strains from all known *H. pylori* biogeographic populations. The gene content and arrangement of the *cag*PAI and its function were highly conserved. Diversity in most *cag* genes consisted in large part of synonymous polymorphisms. However some genes—in particular those that encode proteins predicted to be secreted or located on the outside of the bacterial cell—had particularly high frequencies of non-synonymous polymorphisms, suggesting that they were under diversifying selection. Our study provides evidence that the *cag*PAI was only acquired once and provides an important resource that can guide future research on the biological roles and host interactions of *cag*PAI proteins, including several whose function is still unknown.

## Introduction

*Helicobacter pylori* persistently infects more than one half of all humans, and can cause ulcer disease, gastric cancer, and MALT lymphoma [1]. The *H. pylori cag* pathogenicity island (*cag*PAI) is an intriguing virulence module of this obligate host-associated bacterium [2]–[4]. *H. pylori* strains that possess a functional *cag*PAI are particularly frequently associated with severe sequelae, notably gastric atrophy and cancer [4]–[7]. The *cag*PAI is ∼37 kb long, and contains ∼28 genes [3]. These genes encode multiple structural components of a bacterial type IV secretion system (t4ss) as well as the 128 kDa effector protein, CagA [7]. After *H. pylori* has adhered to a host cell, the Cag t4ss translocates CagA into that cell. CagA is subsequently phosphorylated by host cell kinases and interacts with multiple targets (e.g. SHP-2, Grb2, FAK), profoundly altering host cellular functions [8], [9]. The alterations induced by the *cag*PAI are thought to ultimately contribute to malignant transformation [4], [10], and CagA has been designated a bacterial oncoprotein [11].

*H. pylori* has a high mutation rate, which has resulted in extensive genetic diversity [12], and also recombines frequently with other *H. pylori* [13]. *H. pylori* isolates have been subdivided into distinct biogeographic populations and subpopulations with specific geographical distributions that reflect ancient human migrations [14]–[16]. The global population structure of *H. pylori* is now well understood based on multilocus haplotypes from seven housekeeping genes. However, very little is known about the biogeographic variation of virulence factors, such as the *cag*PAI, nor has the impact of genetic variation on disease outcome and host adaptation been adequately addressed. Previous analyses on the basis of comparative genome hybridization have demonstrated marked differences between biogeographic populations with respect to the *cag*PAI [17]. Microarray analysis of 56 globally representative strains of *H. pylori* revealed that the *cag*PAI was present in almost all strains from some biogeographic populations and subpopulations in Africa and Asia, while it was variably present in other populations [17]. The *ca*gPAI was lacking in all isolates of hpAfrica2, which is distantly related to the other populations [17]. Currently, nine complete *cag*PAI sequences are publicly available [2], [18]–[22], whose isolates belong to hpEurope (7 sequences), hspWAfrica (1) and hspEAsia (1) (see Results), and no sequence data is available for the *cag*PAI in the other six populations and subpopulations where the *cag*PAI is present.

Here we analyze complete *cag*PAI sequences from 38 isolates representing all known *H. pylori* populations and subpopulations and compare their genetic polymorphisms with measures of functional expression. Our data show that the *cag*PAI has shared a long evolutionary history with the *H. pylori* core genome, and displays a remarkable global conservation of gene content, structure and function, with minor exceptions. We provide evidence that the *cag*PAI was acquired by ancestral *H. pylori* in a single event that occurred before modern humans migrated out of Africa. Sequence comparisons identified domains in multiple components of the t4ss that are likely to be under diversifying selection, and these findings can guide future research into the function of t4ss components.

## Results

### Distribution of the *cag*PAI in a global collection of *H. pylori*

**Figure 1:**
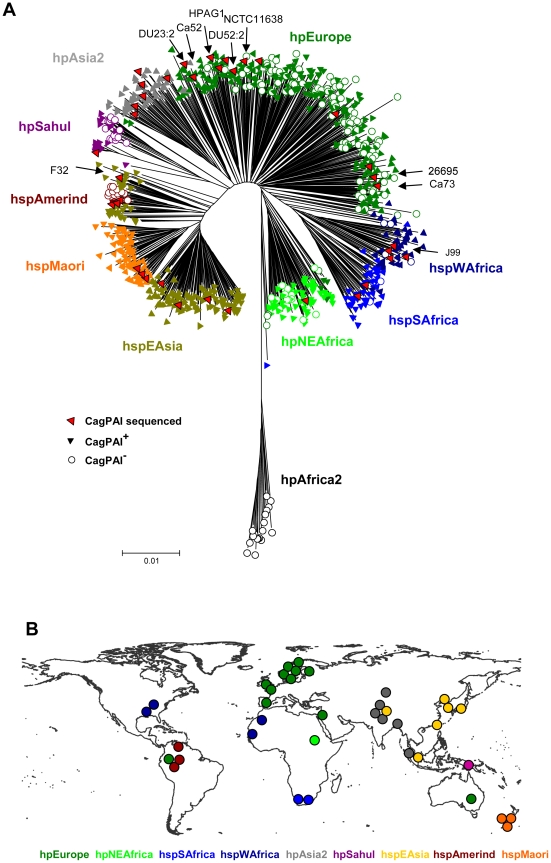
Distribution of the *cag* pathogenicity island in a global collection of *H. pylori* strains from different populations. (A) Neighbor joining (NJ) tree of neutral genetic relatedness of *H. pylori* strains, including information about the presence or absence of the *cag*PAI. The NJ tree was calculated from concatenated sequences of seven housekeeping genes (length 3406 bp) from 877 isolates of *H. pylori* [16] plus 9 additional isolates from which either *cag*PAI sequences [20] or whole genome sequences had been published (indicated by arrows; [2], [18]–[22]. Each strain was scored for presence (filled triangles) or absence (empty circles) of the *cag*PAI based on the results of PCR reactions that span the ends of the *cag*PAI. Population assignments based on Bayesian analyses [15], [16] are indicated by the color coding of symbols that correspond to the labels next to the tree; red symbols indicate all strains whose *cag*PAI sequences are now available, including the 29 strains that have been newly selected for *cag*PAI sequence analysis. (B) Geographic sources of strains whose *cag*PAI sequences are now available. Each dot indicates the source of isolation of one of the 38 *cag*PAI sequences that were analyzed. The dots are color-coded by population or subpopulation as in (A).

In order to define the occurrence of the *cag*PAI in *H. pylori*, we screened a globally representative collection of *H. pylori* isolates from 53 different geographical or ethnic sources [15], [16] (Figure 1). 877 isolates were tested for the presence of the *cag*PAI by a PCR approach. Strains were classified as *cag*PAI-positive if we succeeded in separate PCR amplifications for the 5′ and 3′ ends of the *cag*PAI, or as *cag*PAI-negative if we succeeded in amplifying an empty site with primers from the flanking regions. The *cag*PAI was present in at least 95% of strains assigned to the hpAfrica1 (hspWAfrica plus hspSAfrica), hpEastAsia (hspEAsia, hspMaori) and hpAsia2 populations. In contrast, none of the hpAfrica2 strains possessed the *cag*PAI, and it was only variably present in strains from the populations hpEurope (225/330 strains; 58%), hpNEAfrica (58/72: 81%), and hpSahul (32/49; 65%) or the hspAmerind subpopulation of hpEastAsia (5/18; 28%).

Based on their multilocus sequence typing (MLST) haplotypes, seven strains with published *cag*PAI sequences belong to the hpEurope population (NCTC11638 from Australia [2]; 26695 from England [18]; and DU23, DU52, Ca52, Ca73 [20] and HPAG1 [21] from Sweden). J99 from the U.S.A. [22] belongs to hpAfrica1, and F32 [19] from Japan belongs to the hspEAsia population of hpEastAsia. None of these published *cag*PAI sequences were from strains of the hpNEAfrica, hpSahul, or hpAsia2 populations, from the hpEastAsia subpopulations hspAmerind or hspMaori, or from the hpAfrica1 subpopulation hspSAfrica, although those populations are also potentially important for our understanding of the evolutionary history of *H. pylori*. We therefore selected 29 strains from our global strain collection to supplement these nine published *cag*PAI sequences and provide a globally representative sample of *cag*PAI diversity (Figure 1). These strains included all known biogeographic populations, except for the *cag*-negative hpAfrica2. The entire *cag*PAI, approximately 37 kilobasepairs in length, was sequenced and annotated from each of the 29 strains, either after shot-gun cloning of overlapping long-range PCR products or via direct amplification of multiple, smaller PCR products.

### Conserved synteny and low macrodiversity in the *cag*PAI

**Figure 2:**
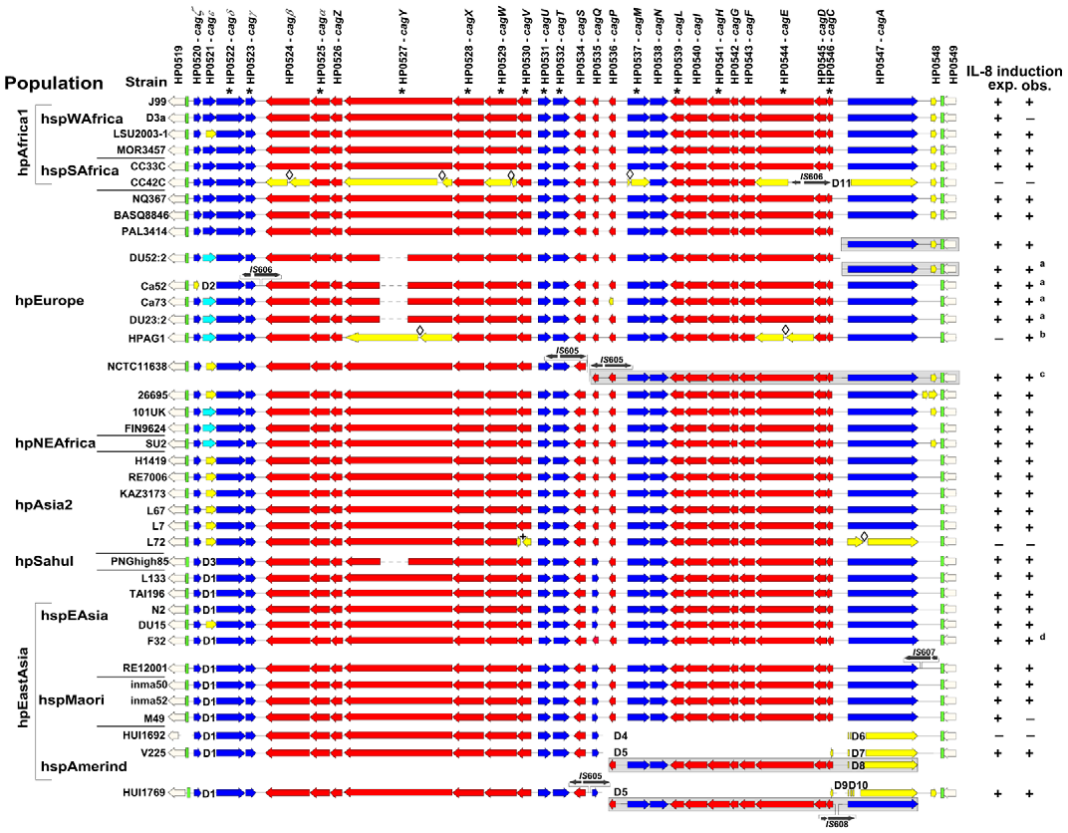
Conservation of the *cag*PAI genetic organization across *H. pylori* biogeographic populations. The sequences were aligned in KODON using the *cag*PAI of strain J99 as a scaffold sequence. Individual isolates are grouped according to biogeographic (sub-)populations. The continuity of the *cag*PAI was disrupted in isolates PAL3414, V225 and HUI1769, and fragments found in secondary locations are displayed in grey-shaded boxes on separate lines. The two *cag*PAI sequences from reference strains J99 and 26695 were extracted from whole genomes. Genes essential for a basic function of the *cag*PAI type IV secretion system (IL-8 induction; [3]) are labeled with an asterisk*. Activity of the Cag t4ss (IL-8 secretion; + or −) was monitored during experimental infection of AGS cells with *H. pylori*. Obs., observed IL-8 secretion; exp., IL-8 secretion expected from the *cag*PAI sequence; red, genes in forward orientation; blue, genes in reverse orientation; light blue, shorter gene version; white, different gene HP521B [20] in this locus; yellow, pseudogenes; black, IS elements; green, *cag*PAI insertion sites. Diamonds: frameshift mutations leading to pseudogenes. Δ followed by numbers 1 through 10 indicate different deletions (manifestation of macrodiversity) and are consecutively numbered as mentioned in the text and Table 1. a,b,c,d: strains not functionally tested in this study possess functional *cag*PAIs according to the following references: a [20]; b [21]; c [2]; d [19].

**Figure 3:**
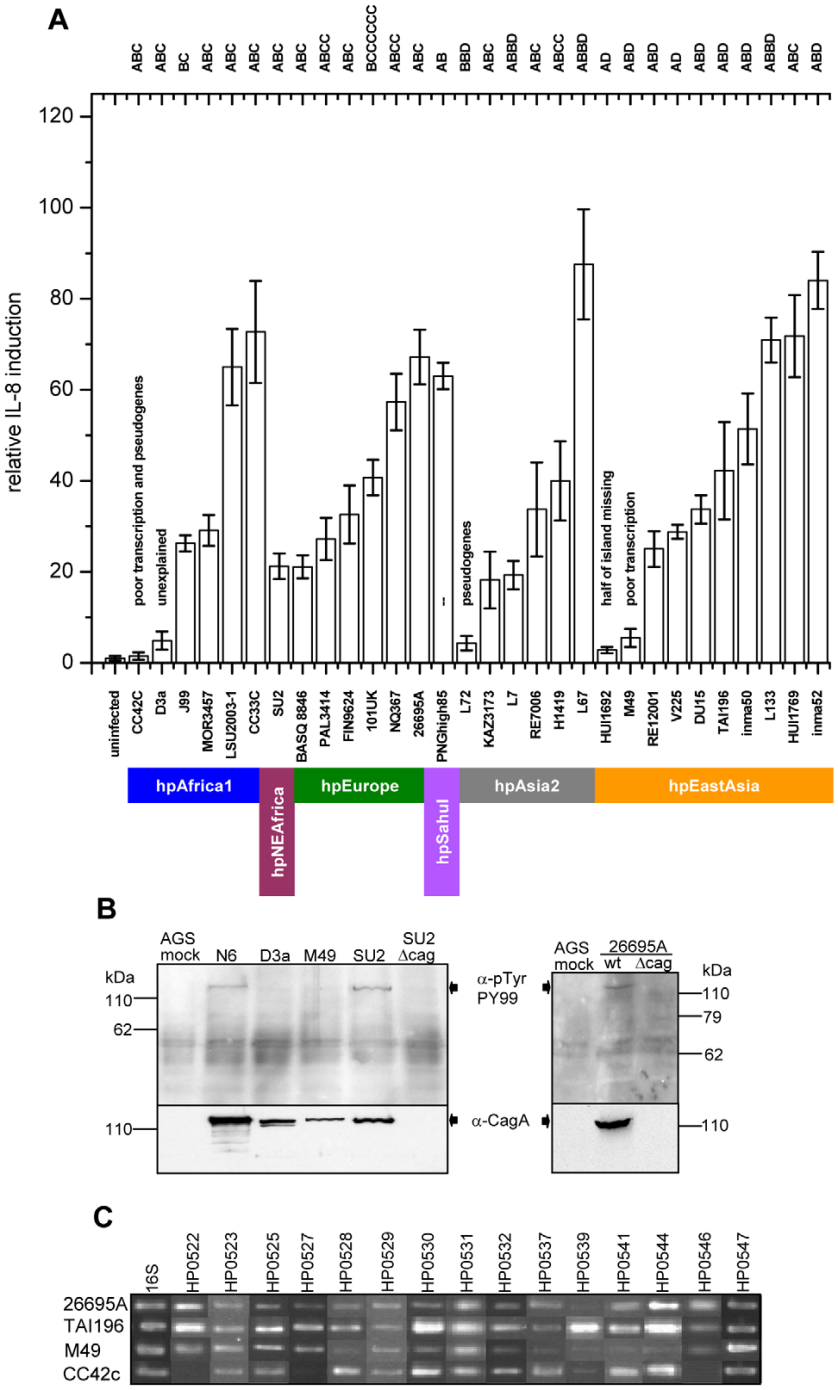
Variability of Cag t4ss function in *H. pylori* strains from different biogeographic populations. (A) IL-8 induction in human gastric epithelial cells by diverse *H. pylori* strains from different biogeographic populations. IL-8 secretion induced at 20 h post infection by live *H. pylori* in gastric epithelial cells (AGS, shown here, and MKN28, data not shown) was determined as a read-out for Cag t4ss activity. The two strains J99 and 26695A, for which entire genome sequences are available, were included as positive controls. CagA EPIYA motifs for each strain are indicated on top of the graph. Exceptions in the genetic integrity of some of the islands and other explanations for an observed loss of functionality are indicated above the single bars. Colored bars designate the population assignments of strains. Coincubation experiments were performed independently at least three times for each strain, with similar results, and one representative experiment, performed in triplicates for each strain, is shown. IL-8 secretion is depicted in relative values, as a multiple of the negative control (mock), which was set to 1. (B,C) Assessing underlying causes of loss of function of *cag*PAIs in some *H. pylori* strains. (B) CagA translocation assays performed after infection of AGS cells with the two selected *H. pylori* strains D3A and M49. These displayed loss of *cag*PAI-related activity in IL-8 release assays. Both strains were unable to translocate CagA into human gastric epithelial cells. Strains SU2, N6, and 26695A wild type (wt) were used as positive controls for CagA translocation. Strains SU2Δ*cag* and 26695AΔ*cag* (isogenic *cag*PAI deletion mutants to SU2 and 26695A) were included as negative controls. (C) transcript amounts of single *cag*PAI genes. 30 strains (4 strains shown here – for complete results see Table S3) were studied using semiquantitative RT PCR for each gene with known function in the Cag t4ss (refer to Table 2 for gene names). Two strains with loss of t4ss function, CC72C, and M49, are shown. TAI196 and 26695A are depicted as positive controls. TAI196, a strain with a high propensity to induce IL-8, shows relatively high transcript amounts for the majority of genes. Strains CC42C and L72 (not shown) which have pseudogenes and lost the ability to induce IL-8, showed low or undetectable transcript amounts for some genes including the pseudogenes. M49 displayed low transcript amounts for a number of essential genes of the t4ss located predominantly in the right half of the *cag*PAI (genes HP0528, and HP0537 to HP0544).

The 38 complete *cag*PAI sequences were compared by pairwise sequence alignments and by a multiple alignment in Kodon relative to the *cag*PAI from J99 used as a scaffold sequence (Figure 2). The general pattern of gene content and gene order (signifying macrodiversity) was similar in most sequences, with only limited variation due to changed synteny or deletions. Synteny changes resulted from genomic rearrangements, horizontal genetic exchange (e.g. replacement of HP0521 by HP0521b), possibly in conjunction with IS (insertion sequence) element insertion, or gene inversions, such as for HP0535. Insertions, deletions, point mutations, frameshift mutations or disruption through insertion elements (Figure S1) were also observed in some of the *cag*PAI sequences, some of which should have resulted in pseudogenes. We therefore tested all strains for their ability to induce interleukin-8 (IL-8) in gastric epithelial cells (Figure 2, Figure 3), as an indicator of PAI function [23]. Most of the strains containing a *cag*PAI were able to induce IL-8, indicating that many of the mutations did not drastically reduce the general function of the *cag*PAI (Table 1).

**Table 1:** Genetic macro- and minidiversity variants (gene order and orientation, gene identity, insertion elements) within the *H. pylori cag*PAI with regard to population assignments.

Frequency	Type	Occurrence (population or strain)	Gene and/or position in J99	IL-8 induction
**Frequent**	Mini-IS*606*a	hspWAfrica (3/4), hpEurope (7/12), hspAmerind (3/3)	41–232 (192 bp)	+
	Mini-IS*606*b	hpAsia2 (6/6), hspEAsia (4/6), hspMaori (3/3)	21300 (140 bp)	+
	Mini-IS*606*c	hpAfrica1 (6/6), hpEurope (8/12), hpAsia2 (6/6), hpNEAfrica (1/1), hspAmerind (3/3)	36969–37098 (130 bp)	+
	Mini-IS*606*d	hspEAsia (6/6), hspMaori (3/3), hpEurope (3/12)	36969–37098 (303 bp)	+
	Inversion	hpEastAsia (11/12)^1^	HP0535	+
	Deletion 2 (Δ2)	hpEastAsia (11/12)	HP0521, 843–1467	+
	Shortened gene	hpAsia2 (6/6), hspEAsia (1/6), hpEurope (2/12), hspWAfrica (1/4)^2^	HP0521	+
	Rearrangement	HspAmerind (2/3)	HP0536 – HP547, 21182–36740	+
	Mini-IS*605*	hpEurope (5/12), hpNEAfrica (1/1), hpAsia2 (1/6)	37003	+
	Replacement	hpEurope (5/12), hpNEAfrica (1/1)^3^	HP0521B, 797–1392	+
**Rare**	Frameshift	CC42C	HP0524, 5166 (+1, 7C → 8C)	−
	Frameshift	CC42C	HP0527, 12851 (+1, 3A → 4A)	−
	Frameshift	CC42C	HP0529, 16416 (+1, 2T → 3T)	−
	Frameshift	CC42C	HP0537, 22392 (−1, 7A → 6A)	−
	Frameshift	HPAG1	HP0527, 11326 (−1, 6A → 5A)	+^4^
	Frameshift	HPAG1	HP0544, 30118 (−1, 3A → 2A)	+^4^
	Frameshift	L72	HP0547, 34034 (−1, 7A → 6A)	−
	Stop codon	L72	HP0530, 16932, CGA → TGA	−
	Mini-IS*605*	MOR3457	17505	+
	Mini-IS*606*e	26695	36969–37003 (35 bp)	+
	IS*605*	NCTC11638, HUI1769^5^	20345	+
	*IS*606	Ca52	3605	+
	IS*606*	CC42C	30450–33503	−
	IS*607*	RE12001^6^	37718	+
	IS*608*	HUI1769	32724	+
	Rearrangement	NCTC11638^7^	HP0535 – HP0549, 20345	+
	Rearrangement	DU52:2, PAL3414	HP0547 – HP0549, 33360	+
	Deletion 1 (Δ1)	Ca52	618–1467	+
	Deletion 3 (Δ3)	CC42C	30450–33503	−
	Deletion 4 (Δ4)	HUI1692	21182–33406	−
	Deletion 5 (Δ5)	V225, HUI1769	21182–32492	+^8^
	Deletion 6 (Δ6)	HUI1692	33593–34247	+
	Deletion 7 (Δ7)	V225	33596–34318	+
	Deletion 8 (Δ8)	V225	33450–34247	+
	Deletion 9 (Δ 9)	HUI1769	32669–33116	+
	Deletion 10 (Δ10)	HUI1769	33692–34100	+

a, b, c, d, e represent different genetic variants of mini-IS*606*; mini-IS*606* variants c, d and e were collectively referred to as “remnant *IS*606* within the *cag* right end segment” by Kersulyte *et al*. [26].

1 Also in 8/11 strains from Japan [19]. The inversion encompasses a total of 1230 bp that are present in hpAsia2 and consists of HP0535 plus 483 bp of upstream and 381 bp of downstream flanking non-coding DNA. The homologous stretch in J99 contains flanking non-coding DNA stretches of 50 bp upstream and 160 bp downstream that are replaced by 490 bp and 460 bp, respectively, in hpAsia2 strain KAZ3173 (see Figure S1).

2 357 bp versus 659 bp for HP0521 in J99.

3 Also in 34/63 strains from Sweden [20].

4 IL-8 induction is according to data published by Oh *et al*. [21]. However, HP0527 and HP0544 possess frameshift mutations that would normally prevent induction of IL-8.

5 Found in 1/95 additional strains from a global survey (this study) and 11/40 strains from Italy [2].

6 in 1/95 additional strains from a global survey (this study).

7 also found in 4/40 strains from Italy [2].

8 Deletion would prevent IL-8 induction. IL-8 induction is observed because of the presence of HP0536 – HP0547 in another genomic location.

### Fixed and transient variants in *cag*PAI sequence organization

Most new mutations are deleterious, whether associated with single nucleotide polymorphisms, mobile elements or genomic rearrangements, and will be removed by purifying selection. However, mutations without a drastic effect on fitness, so-called neutral or nearly neutral mutations, can remain as rare variants within a population for long time periods. The vast majority of such mutations remain at low frequency until they are (usually) lost due to genetic drift. Rare neutral mutations can become more frequent over time, or even become fixed, also due to genetic drift [24]. Still other mutations are under positive selection. These rapidly become frequent or fixed due to Darwinian selection. In isolated clonal populations, Muller's ratchet can even result in some deleterious mutations rising to high frequency [25] and the same is true of extreme bottlenecks, which can fix deleterious mutations immediately. These basic evolutionary principles indicate that the demographies of rare versus frequent mutations differ and should be examined separately.

### Frequent variants

A number of frequent *cag*PAI macrodiversity variants were found, some of which were present in all isolates of at least one sub-population, or almost all isolates (Table 1). These included insertion events due to one of three variants of IS*606* [26] or of a mini-IS*605* insertion [27], [28], an inversion of gene HP0535 plus its flanking non-coding DNA, a deletion of either the complete HP0521 ORF (Δ2; Figure 2) or part of that ORF, or the replacement of HP0521 by the unrelated ORF HP0521B (Figure 2, Table 1). Additionally, most of the 3′ (right) half of the *cag*PAI is lacking in all three hspAmerind strains due to one of two similar 11.2 kb deletions with distinct 3′ ends (Δ4, Δ5; Figure 2). These large deletions terminate within HP0546, and are associated with a second (intergenic) deletion of 410 bp or a 620 bp deletion that terminates within the N-terminal part of HP0547 (*cagA*). In strains V225 and HUI1769, a copy of the deleted segment plus the HP0546 and HP0547 ORFs have translocated to a separate, currently unidentified, location of the chromosome, leaving a shortened version of HP0546 at the original location (Figure 2). It is interesting to note that IL-8 induction was not eliminated by any of these frequent mutations (Figure 2, Figure 3, Table 1), suggesting that they are not deleterious to *cag*PAI function, and might be neutral or even under positive selection.

### Rare variants

Rare variants were present in only one or two strains, are probably transient, and will tend to disappear during genetic drift [29]. The rare variants included frameshift mutations in multiple ORFs within three single isolates (CC42C, HPAG1 and L72) and IS elements (mini-IS*605*, IS*605*, IS*606*, IS*607* or IS*608* [26]) that have integrated at distinct locations in 7 other isolates (Table 1; Figure S1). Our dataset consisted of only 38 isolates, and it was possible that these rare mutations might be more widely distributed. We therefore screened 95 other globally representative strains for the presence of IS*605*, IS*606*, IS*607* or IS*608* at those locations, but only identified two additional strains with IS element insertions, one each for IS*605* (MOR3055 – hspWAfrica) and IS*607* (BASQ9523 – hpEurope) (data not shown). Thus, strains carrying these particular insertion mutations really are rare.

We also found two rare, distinct genomic rearrangements (Table 1). One of these was in strain NCTC11638 from Australia and has been reported previously [2]. It splits the *cag*PAI between ORFs HP0534 and HP0535 into two segments, one of which is translocated elsewhere in the genome, and is distinct from the split of the *cag*PAI in the hspAmerind strains. Previous analyses identified the same rearrangement in 4/40 strains from Italy [2], but it was not found in any of the other 38 *cag*PAI sequences analyzed here nor in any of the 95 other, globally representative strains that we investigated by PCR. The other rearrangement separated HP0547 (*cagA*) through HP0549 plus flanking DNA from the rest of the *cag*PAI. It has been previously described for two hpEurope strains from Sweden and one from Australia [20]. We found the same pattern in a fourth hpEurope strain isolated in Palestine (PAL3414). Both of these rearrangements were present in less than 5% of isolates.

The 17 rare mutations were identified in a total of 12 isolates. Only three of those, CC42C, HUI1692 and L72, did not induce IL-8, indicating that the majority of the rare sequence changes also did not cause a severe loss of *cag*PAI function. This observation is compatible with most of the rare mutations being selective neutral or near-neutral.

### Genomic decay

Three overlapping small deletions (Δ1, Δ2, Δ3) that removed the HP521 ORF were found in all but one hpEastAsia isolate, one hpEurope isolate and the hpSahul strain (Figure 2; Table 1), but those did not abolish *cag*PAI function (see above). Eight other deletions were found in four individual strains (Figure 2). Two of these isolates were unable to induce IL-8: CC42C (hspSAfrica) contains multiple frameshift mutations and an insertion of IS*606* as well as deletion Δ11, which removes part of *cagA* (HP547). Δ4 and Δ6 deleted half of the *cag*PAI in hspAmerind strain HUI1692. The *cag*PAI is clearly decaying in both CC42C and HUI1692. In contrast, although deletions Δ5 and Δ7–Δ10 also removed large parts of the *cag*PAI in hspAmerind strains V225 and HUI1769, these deletions occurred in a segment that has been duplicated to a separate location (see above) and these two isolates remain able to induce IL-8. Thus, with one exception (Δ1), these deletions are rare and seem to be associated with accelerated decay of non-functional *cag*PAI genes. In addition, the *cag*PAI in non IL-8-inducing strain L72 also contained one frameshift and one premature stop codon in a coding region, and seems to be undergoing decay.

### Signatures of selection within individual *cag*PAI genes

Darwinian selection for variation in coding regions can also be exerted at the nucleotide or protein level. We therefore analyzed sequence polymorphisms (microdiversity) in individual *cag*PAI genes for traces of such selection (Materials and Methods). Similar to housekeeping genes [30], almost all alleles of each *cag*PAI ORF were unique to one isolate among the 38 strains. Exceptionally, we identified duplicates of a single allelic sequence in six genes; in each case, the strains possessing the duplicate alleles were from a common population (Table S4). Occasional duplicate alleles within populations have also been described for housekeeping genes [30] and are considered to represent homologous recombination. Again, similar to housekeeping genes, most *cag*PAI genes seemed to be under purifying selection because their *Ka*/*Ks* ratios were ≤0.2 (Table 2). However, five genes (HP0534-0535, HP0538, HP0546-0547) showed signs of positive or diversifying selection because their overall *K*a/*Ks* ratios were greater than 0.2; of these, *cagA* (HP0547) had the highest proportion of non-synonymous polymorphisms (*Ka/Ks*  = 0.45). However, *Ka/Ks* ratios are relatively insensitive indicators of Darwinian selection, which can act at the level of single protein epitopes or conformational domains. We therefore used a Bayesian method (PAML/CODEML [31]) to search MLST and *cag*PAI genes for codons that might be under diversifying selection (indicated by ω >1). Only two of the seven MLST housekeeping genes (*trpC*, *yphC*) contained an appreciable frequency (3.9%; 5.3%) of codons with posterior probabilities of ω >1 being above 0.95 (Table 2). In contrast, >5.3% of the codons matched this criterion in 10 of the 28 *cag*PAI ORFs (Table 2), including four of the five ORFs with high overall *K*a/*K*s ratios (HP0535, HP0538, HP0546, HP0547).

**Table 2:** Sequence diversity, *K*_s_/*K*_a_ ratios, and codons under diversifying selection in *cag*PAI and housekeeping genes (37 strains).

Gene no. in strain 26695	Gene name	Component of type IV secretion system	Mean sequence diversity (π)	K_a_	K_s_	Ratio K_a_/K_s_	No. of codons	Codons under diversifying selection (ω>1) (PAML)		r *
								Number	%	
HP0520^§^	*cagζ*	u	0.030	0.016	0.084	0.190	115	10	8.70	0.36
HP0522	*cag*Δ	u	0.047	0.019	0.147	0.131	481	9	1.87	0.72
HP0523^§^	*cagγ*	VirB1	0.089	0.036	0.279	0.127	169	9	5.33	0.45
HP0524	*cagβ*	VirD4	0.041	0.007	0.164	0.045	748	4	0.53	0.64
HP0525	*cagα*	VirB11	0.025	0.005	0.124	0.044	330	2	0.61	0.71
HP0526	*cagZ*	u	0.021	0.010	0.065	0.148	199	8	4.02	0.64
HP0527^§^	*cagY*	VirB10	0.049	0.017	0.097	0.171	2797	433	15.48	0.62
HP0528	*cagX*	VirB9	0.024	0.006	0.092	0.068	522	10	1.92	0.74
HP0529	*cagW*	VirB6	0.025	0.008	0.080	0.102	536	17	3.17	0.25
HP0530	*cagV*	VirB8	0.024	0.006	0.093	0.066	252	9	3.57	0.50
HP0531	*cagU*	u	0.032	0.012	0.095	0.123	218	4	1.83	0.60
HP0532	*cagT*	VirB7	0.026	0.006	0.101	0.061	280	9	3.21	0.61
HP0534	*cagS*	u	0.025	0.015	0.070	0.210	199	4	2.01	0.68
HP0535^§^	*cagQ*	u	0.061	0.039	0.153	0.254	101	10	9.90	0.38
HP0536^§^	*cagP*	u	0.031	0.011	0.079	0.138	117	7	5.98	0.43
HP0537	*cagM*	u	0.026	0.008	0.097	0.078	376	4	1.06	0.52
HP0538^§^	*cagN*	u	0.034	0.021	0.081	0.263	306	34	11.11	0.57
HP0539^§^	*cagL*	VirB5	0.032	0.016	0.087	0.185	237	21	8.86	0.17
HP0540^§^	*cagI*	u	0.032	0.017	0.087	0.196	381	23	6.04	0.40
HP0541	*cagH*	u	0.027	0.010	0.087	0.110	370	10	2.70	0.26
HP0542	*cagG*	u	0.029	0.010	0.097	0.102	143	0	0.00	0.57
HP0543	*cagF*	u	0.029	0.014	0.095	0.143	268	10	3.73	0.52
HP0544	*cagE*	VirB3/VirB4	0.026	0.005	0.103	0.049	984	9	0.91	0.62
HP0545	*cagD*	u	0.039	0.016	0.122	0.134	209	5	2.39	0.38
HP0546^§^	*cagC*	VirB2	0.051	0.031	0.112	0.277	116	7	6.03	0.33
HP0547^§^	*cagA*	effector	0.088	0.067	0.150	0.448	1389	381	27.43	0.40
Merged *cag*PAI genes			0.040	0.012	0.115	0.106	-	-	-	0.65
HP1134	*atpA*		0.021	0.002	0.111	0.016	209	1	0.48	0.61
HP0177	*efp*		0.032	0.001	0.141	0.007	136	0	0.00	0.54
HP0142	*mutY*		0.058	0.018	0.198	0.089	140	0	0.00	0.61
HP0620	*ppa*		0.028	0.004	0.117	0.036	132	0	0.00	0.48
HP1279	*trpC*		0.069	0.030	0.204	0.149	152	6	3.95	0.57
HP0071	*ureI*		0.029	0.007	0.102	0.066	195	0	0.00	0.40
HP0834	*yphC*		0.042	0.015	0.140	0.107	170	9	5.29	0.64
Merged hk genes			0.041	0.011	0.141	0.076	-	-	-	-

Mantel test (r*) between matrices of individual *cag*PAI genes versus concatenated housekeeping genes.

r* Pearson correlation coefficient of p-distance matrices from individual genes versus concatenated housekeeping genes. R values for the housekeeping genes were calculated from matrices of concatenated sequences jackknifing from the respective gene. ^§^ *cag* genes predicted to be under diversifying.selection (p>95% in > = 5.3% of codons in PAML). u  =  genes of partly or completely undefined function. Total number of codons per gene refers to alignment length used for PAML.

**Figure 4:**
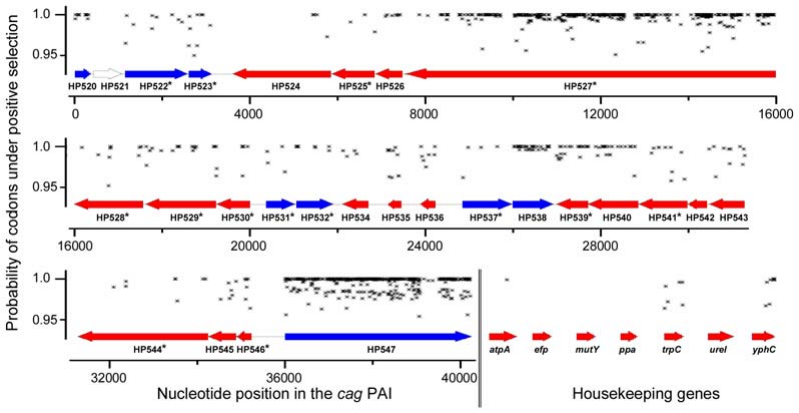
Sliding window map of maximum likelihood analysis of codons to be under diversifying selection for complete *cag*PAIs and housekeeping genes. Codons calculated by CODEML (model M3) to have a high likelihood p>95% of being under diversifying selection in each gene of the *cag*PAI or housekeeping genes of all analyzed strains are highlighted by black symbols.

We also tested eleven *cag*PAI ORFs, including nine with high frequencies of codons under selection according to PAML, and two with lower frequencies (HP0524, HP0525) with a second Bayesian program, OmegaMap [32], [33], which unlike PAML also takes into account the occurrence of recombination (ρ) between different alleles (Table S5). OmegaMap detected fewer codons with high probabilities of positive selection, but the codons that it identified often overlapped with codons that had been identified as being under positive selection by PAML (Table S5). Finally, we employed a sliding window along codons of PAML posterior probabilities of ω to identify clusters of sites with signs of diversifying selection (Figure 4). The combination of three forms of analysis (criteria: Ka/Ks >0.2, or likelihood of at least 95% for ω >1 in ≥5.3% of codons, or at least two clusters of two or more adjacent amino acids (aa) predicted under diversifying selection in PAML) identified 13 *cag*PAI genes that are likely to have evolved under diversifying selection: HP0520, HP0522, HP0523, HP0527, HP0528, HP0534, HP0535, HP0536, HP0538, HP0539, HP0540, HP0546 and HP0547. Of these, functions or structural contributions are known only for HP0523 (*virB1*), HP0527 (*virB10*), HP0539 (*virB5*), HP0546 (*virB2*) and HP0547 (*cagA*) [7], [34]–[38]. The percentage of codons with high likelihood of positive selection was highest in *cagA* (26.9%), followed by *cagY* (15.5%) and a gene of unknown function, *cagQ* (HP0535; 9.9%) (Table 2).

In addition to a high frequency of putative codons under diversifying selection, HP0527 (*cagY*) and HP0547 (*cagA*) also exhibited variable gene lengths. This was due to variable numbers of repetitive modules within the genes, as previously reported [35], [39]. In the CagA protein, the number of phosphorylation sites (C-terminal EPIYA repeat motifs) differed, as did the types of these repeats (Figure 3). As previously described [39], the third EPIYA motif of CagA was type D in most (13/17) Asian strains whereas type D was not found in isolates from any other population. This reflected the preponderance of type D EPIYA in isolates assigned to the hpEastAsia and hpAsia2 populations. If the EPIYA type D motif were ancestral in Asian populations, this finding might reflect horizontal acquisition of *cagA* by the four exceptional Asian strains from Western strains. Homologous recombination involving the *cag*PAI has also been reported in isolates from Mestizos in Peru [40] and might reflect selection due to functional differences that are related to ethnic specificity.

### Comparison of *cag*PAI and housekeeping gene phylogeny

**Figure 5:**
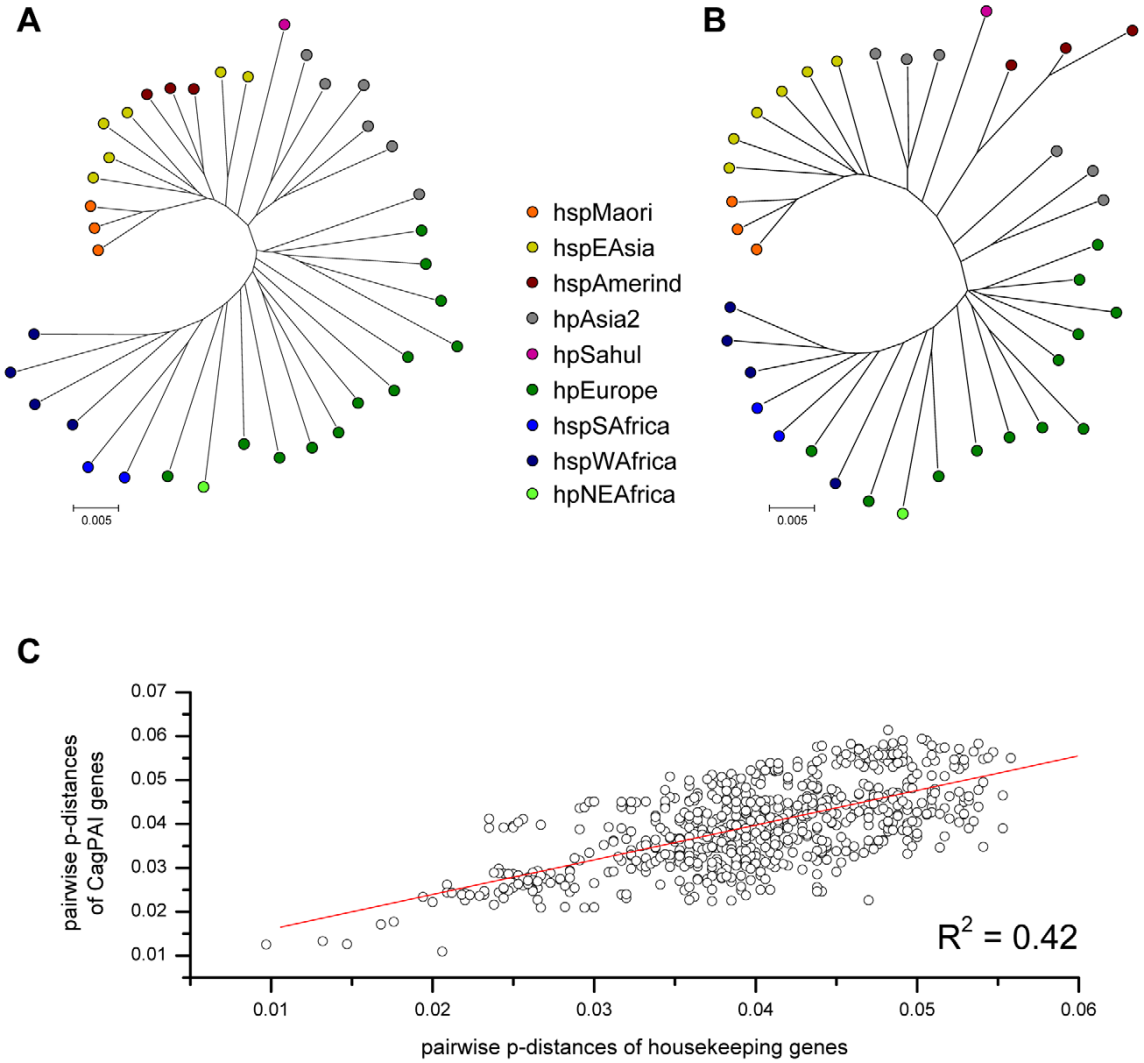
Pairwise correlation of genetic distances and phylogeographic diversity between *H. pylori* housekeeping genes and concatenated *cag*PAI genes. (A) neighbor-joining (NJ) tree analysis of concatenated housekeeping genes for all strains, whose complete *cag*PAIs were analyzed. (B) NJ tree analysis of concatenated *cag*PAI genes for all strains. (C) Mantel comparison of pairwise genetic distances in housekeeping genes and *cag*PAI genes.

We next asked whether the phylogeny of cagPAI genes was similar to that of housekeeping genes. Concatenated sequences of the *cag*PAI genes yielded a tree (Figure 5B) that is very similar to the tree based on a concatenate of the seven MLST housekeeping genes (Figure 5A). Similarly, matrices of pairwise genetic distances of the concatenated *cag*PAI genes were highly correlated with corresponding matrices of pairwise distances of concatenated housekeeping genes (R = 0.65, p<0.001) (Figure 5C). These data show that 42% of the variance among *cag*PAI genes can be attributed to a linear relationship with housekeeping genes. The correlations for individual *cag*PAI genes ranged from R = 0.17 to R = 0.74 (Table 2). While most *cag*PAI genes thus fell into the range observed for the individual housekeeping genes (0.46 to 0.69), the correlations were lower for particular *cag*PAI genes (e.g. *cagL*, R = 0.17), which might reflect selection and/or recombination between *cag*PAIs from different bacterial populations. These observations indicate a generally similar genealogy of *cag*PAI and housekeeping genes, which would imply that the *cag*PAI has accompanied *H. pylori* since before human migrations out of Africa some 60,000 years ago [17]. In agreement, the genetic diversity of the *cag*PAI genes per population decreased significantly with distance from Northeast Africa (data not shown).

### *cag*PAI sequence variation and type IV secretion system function

Only five of the strains tested here were not able to induce IL-8 (Figure 3). The same five strains did not translocate CagA into AGS cells, a second marker of t4ss function (Figure 3B). For three of the five strains (CC42, L72 and HUI1692), a lack of function can be explained by sequence features of coding sequence (CDS) decay. The *cag*PAI of CC42C contains multiple pseudogenes, some of which are crucial for t4ss function [3]. Half of the *cag*PAI including numerous essential t4ss genes is lacking in strain HUI1692. For strain L72, a point mutation results in a premature stop codon in gene HP0530, which is essential for t4ss function. In contrast, the *cag*PAI sequences did not offer obvious explanations for the lack of induction of IL-8 by strains M49 and D3a. We therefore investigated the transcript abundance of all 14 genes involved in IL-8 induction and of *cagA* for 28 sequenced strains as well as for the reference strains 26695A and J99 (Figure 3C; Table S3). The inability of strain M49 to induce IL-8 can be accounted for by very low transcript levels for 7/15 *cag*PAI genes (Figure 3C; Table S3); the cause of this low transcription is unknown. However, we are unable to explain the inability of strain D3a to induce IL-8, because it was not impaired in *cag*PAI transcription (Table S3). We are also not readily able to explain the considerable variation of transcript levels among the other strains that did induce IL-8 (Table S3), except that it did not correlate with the macrodiversity patterns described above (data not shown).

Similar to the variable transcript levels, the levels of IL-8 induction also varied dramatically (Figure 3). This variation did not correlate with strain assignments to biogeographic populations or with the type and number of EPIYA motifs within CagA (Figure 3A; [39]). Nor did they correlate with quantitative values for adhesion of the strains to AGS or MKN28 gastric epithelial cells (data not shown).

## Discussion

Since its discovery in 1996 [2], the *cag*PAI has probably been the most intensively studied segment of the *H. pylori* genome. The virulence functions of the Cag t4ss and its translocated effector, CagA, have been investigated in great detail, and numerous studies have correlated *cag*PAI-associated polymorphic markers with disease risk. However, all these studies focused on one or only few genes within the *cag*PAI (such as *cagA*), and were performed with strains from one or few geographic regions. We therefore anticipated that a comparative analysis of complete *cag*PAI sequences from a globally representative and well characterized collection of strains would provide valuable information about the evolutionary history of the *cag*PAI and its variability within a phylogeographic context. The complete *cag*PAI sequences of 29 strains were determined and combined with 9 published complete sequences to yield a large and comprehensive dataset of *ca*gPAI diversity, which was analysed at the levels of both macrodiversity (differences in gene content, synteny and function), and microdiversity (sequence polymorphisms).

### Phylogeographic implications of *H. pylori cag*PAI diversity

It has previously been noted from limited samples that different populations of *H. pylori* differ in the frequency of possession of the *cag*PAI [14], [17]. Our data on 877 isolates from all known *H. pylori* populations and subpopulations provide unambiguous evidence for this variability. Carriage of the *cag*PAI varies from almost universal presence in hpEastAsia and hpAfrica1 through intermediate presence (hpEurope) to complete absence (hpAfrica2) (Figure 1). The *cag*PAI is also absent in the related species *H. acinonychis* [17], which resulted from a host jump from humans to large felines [41]. The absence of the *cag*PAI from hpAfrica2 and *H. acinonychis* has been interpreted as the ancestral state, i.e. *H. pylori* acquired this genomic island by horizontal gene transfer from an unknown source after *H. pylori* had established itself in humans [17]. But when was it acquired, and on how many occasions?

The data presented here indicate that the *cag*PAI was only acquired once because its microdiversity correlated with microdiversity within housekeeping genes (Figure 5). That acquisition was prior to 60,000 years ago, the time when *H. pylori* accompanied modern humans during their migrations “out of Africa” [16], because *cag*PAI sequence microdiversity diminished with distance from North East Africa. An important implication of this conclusion is that, with the exception of hpAfrica2, the variable presence of the *cag*PAI in *H. pylori* populations usually reflects secondary loss, rather than inheritance of the ancestral virgin state.

### Macrodiversity versus fitness and function

Previous analyses have shown that strains that circulate within the same communities, and even within the same stomach, can be mixed in respect to possession of the *cag*PAI [30]. This observation indicates that *cag* positive bacteria do not outcompete *cag* negative bacteria in all environments. Nevertheless, our data support the inference [17] that a functional *cag*PAI provides a fitness advantage to *H. pylori* in most human populations: macrodiversity variants that inactivated t4ss function through deletions or insertion of IS elements were rare, whereas macrodiversity variants that were frequent did not affect t4ss function. For instance, shortening, complete loss or replacement (by HP0521b) of gene HP0521 was observed in almost all populations but this did not reduce *cag*PAI functionality, suggesting that this gene is not important for t4ss functions. Similarly, the genetic organization of the *cag*PAI was in general strongly conserved, and insertion elements did not play a decisive evolutionary role for the *cag*PAIs, unlike previous conclusions [2]. Even separation of the *cag*PAI in two parts did not lead to loss of function, except when a deletion was involved.

### *cag*PAI t4ss microdiversity and signatures of positive selection

High variation at the level of sequence microdiversity was found along the *cag*PAI, but this is also true of housekeeping genes, and might possibly result from the high frequencies of mutation and recombination in *H. pylori* [14], [16]. However, unlike most housekeeping genes, multiple *cag*PAI ORFs showed signs of Darwinian diversifying selection, as indicated by higher *Ka*/*Ks* values and codon-based analyses, which identified specific amino acids or regions of particularly high non-synonymous diversity in 13 *cag*PAI genes (Figure 4, Table 2). In the following we attempt to interpret these measures of selection by mapping them onto known components including structural features of the t4ss encoded by the *cag*PAI.

**Figure 6:**
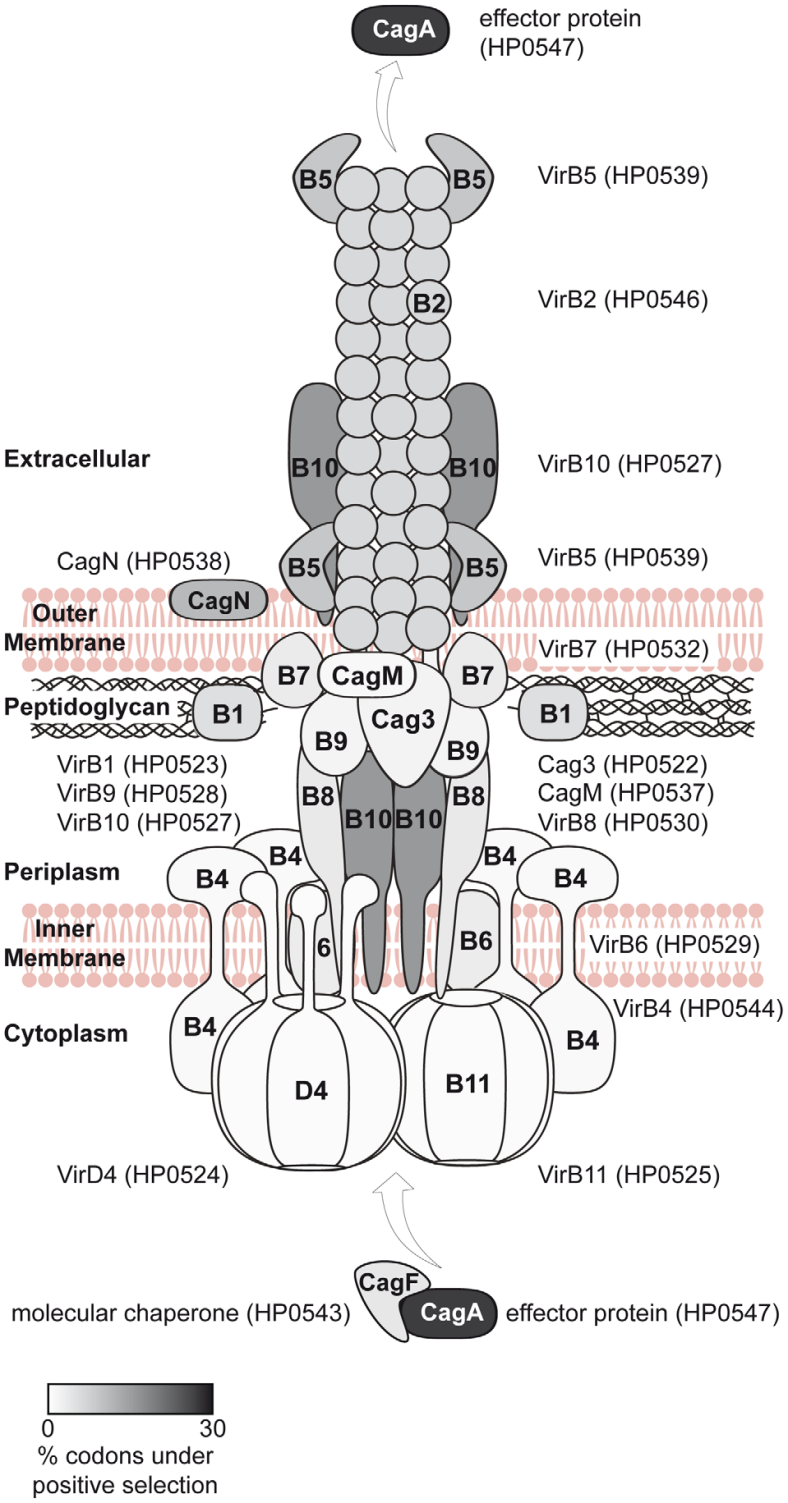
Model of the Cag t4ss of *H. pylori*, highlighting diversifying selection on outer and secreted components of the t4ss apparatus. Each defined component of the *cag*PAI-encoded secretion system was shaded in grey according to averaged probability values, indicating the proportion of amino acids likely to be under diversifying selection for each individual protein; the probability values were calculated for each gene by the software CODEML (Table 2). 10 *cag*PAI genes which do not participate in the structure or are of unknown function are not included in the model. The model of the Cag t4ss is based on [3], [48]–[50], [61], [62].

Seventeen of the *cag*PAI genes are essential for the known t4ss functions (IL-8 induction, CagA translocation [3]), of which 12 have been characterized in structural or functional terms (*virB1,2,4,5,6,7,8,9,10,11* and *virD4* orthologs, *cagA*). In Figure 6, we present a schematic structural model of the *cag*PAI t4ss apparatus including all known structural Cag proteins plus the effector CagA. Different shades of grey indicate the proportion of amino acids which are likely to have undergone diversifying selection according to PAML.

**Figure 7:**
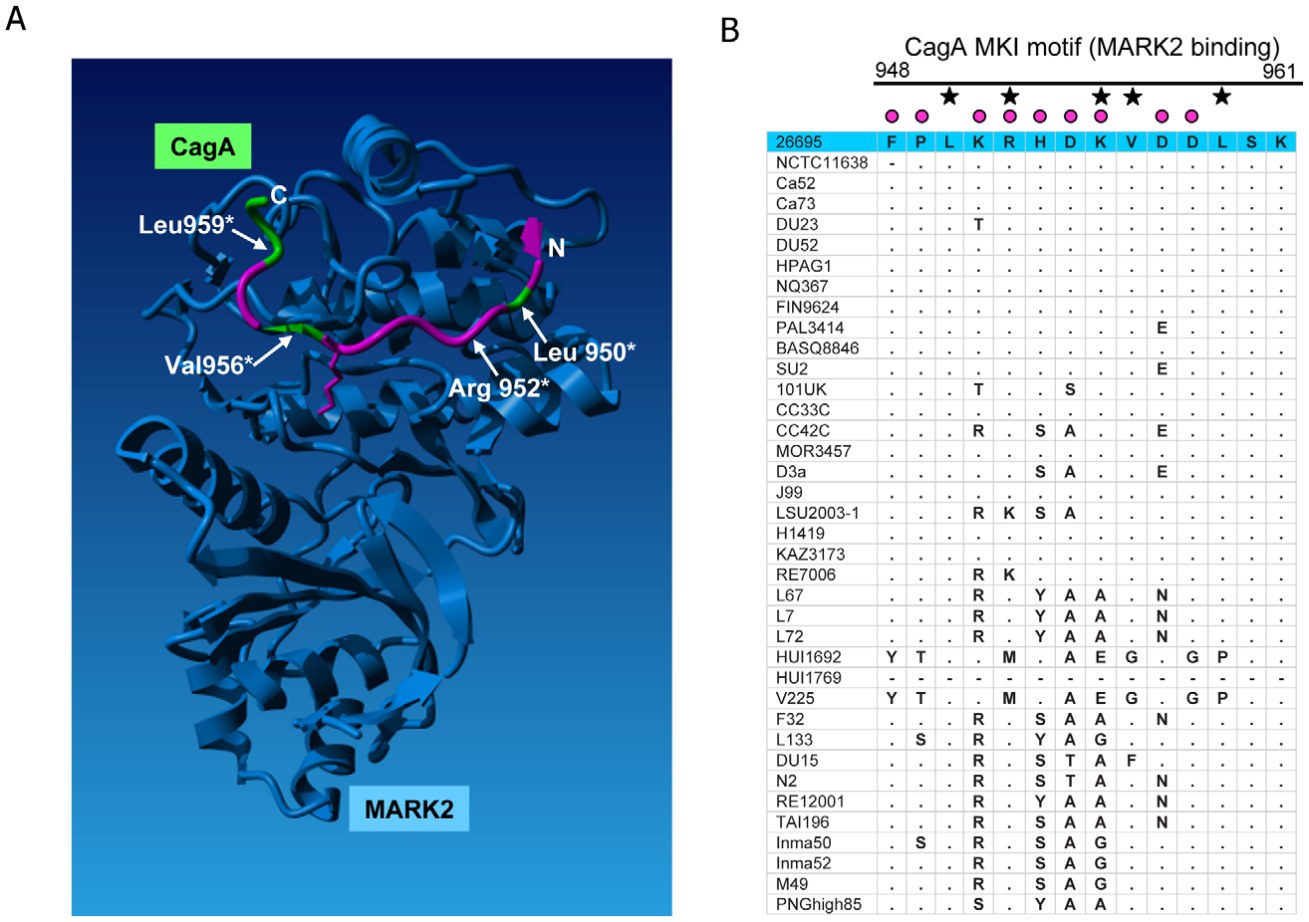
Diversifying selection in the MARK2 kinase binding domain of CagA. (A) amino acids (aa) in CagA predicted to be under diversifying selection (PAML, Model 3) were mapped onto the crystal structure of a short peptide within the CagA C-terminal domain (aa 948 to aa 961, MK1 peptide; aa under positive selection colored in pink, aa not predicted to be under positive selection colored in green), in complex with its interaction partner, the human kinase MARK2 [44]. Four residues critically involved in this interaction (Leu950, Arg952, Val954, Leu959) are labelled. Several amino acids involved in this interaction (e.g. Arg952 and Lys955, Ref. 44) are predicted to be under diversifying selection and are highly variable in our global strain collection. (B) amino acid alignment of the MARK2 binding region in the analyzed global strain collection. Black asterisks: amino acids involved in MARK2 binding. Pink dots: amino acids predicted to be under positive selection. Small dots in alignment: residue identical with reference strain 26695 (blue line on top). Hyphen: aa missing in respective strain.

### CagA

The translocated effector protein CagA (HP0547), which interacts with various host proteins [42], had the highest proportion of such amino acids of the entire *cag*PAI. These were distributed along its entire length, suggesting functional adaptation or modulation. CagA binds to host cell integrins [42] and is translocated into host cells by the *cag*PAI t4ss. Within the host cell, individual domains of CagA interact with intracellular proteins such as SH-2 proteins and protein kinases (e.g. Src, Abl [19], MARK2/PAR1b kinase family [7], [9]). These interactions render it potentially subject to diversifying or positive selection due to host polymorphisms which could even result in modified host protein interactions. A prominent example of amino acid diversity noted previously are the EPIYA motifs in the C-terminal half of CagA, which differ between Asian (hpAsia2; hpEastAsia) (type D) and all other populations [43]. The D type EPIYA repeat binds SHP-2 phosphatase more avidly than other types [19]. A clear bipartite “Eastern”/“Western” separation in the present global dataset was not only observed in phylogenetic trees based on the C-terminal half of CagA containing the divergent EPIYA repeat motifs, but also in its less well-characterized N-terminal moiety. Interestingly, CagA from the ancient and isolated hpSahul population [15] localised in between the Eastern and Western type CagA clusters (not shown).

The global strain selection provided further evidence of functional adaptation in a different CagA motif. Recently, structural analyses of a second CagA subdomain (CM domain, aa 885 to 1005) in complex with its interaction partner from the human host, the cellular kinase MARK2, were performed [44]. This analysis revealed the crucial contribution of specific residues in CagA (MKI motif; [44]) to the physical interaction with the kinase. The short CagA peptide that could be mapped in the cocrystal (Phe_948_–Lys_961_) is characterized in our strain collection by high amino acid variability (Figure 7A and 7B). Superposition of the amino acids under selection (according to PAML) onto the structure of the peptide [44] revealed that all but five of the 14 amino acids in this MARK2 binding domain of CagA have a high posterior probability of being under diversifying selection (Figure 7A). Interestingly, Arg_952_ and Val_956_, which both strongly influence MARK2 binding [44], have a likelihood of 1.0 and 0.81, respectively, of being under positive selection whereas two other MARK2 binding residues, Leu_950_ and Leu_959_, were not under diversifying selection. This result suggests that, although some specific MARK2 binding sites in CagA do have a lower propensity of being under positive selection, the binding strength of CagA to MARK2 can still be influenced by *H. pylori* protein variation, indicative of functional fine-tuning. These predicted functional implications of global variation in the MKI motif are in agreement with an earlier study by Lu et al. [9] who observed differences in CagA PAR1b binding and function when they exchanged two Western and Eastern phylogeographic variants of the CagA MARK2/PAR1b binding region within CagA chimeras. We therefore expect that other regions of CagA that are under selection (Figure 4) also warrant detailed structural and functional analyses. The observed CagA diversity, which is proposed to allow functional fine-tuning, may not only be associated with different host ethnicities but also with niche-dependent intrahost diversification during long-term colonization (e.g. stomach antrum *versus* corpus) [45], [46].

### Other *cag* genes

A prior general comparison of component diversity in type III and IV secretion systems from different bacterial species [47] found that core structural proteins located in the bacterial cytoplasm or the inner membrane exhibit significantly lower diversity than do structural proteins exposed on the surface of the bacteria or secreted effector proteins [47]. Two well-characterized *cag* genes whose gene products are exposed on the cell surface have experienced strong selection: *cagY* (HP0527), which encodes a VirB10 ortholog that is a structural component of the *cag*PAI t4ss [36], and *cagC* (HP0546), which encodes a VirB2 pilin subunit ortholog [35], [38]. CagY is under selection due to host antibodies and/or direct host interactions [35], [36]. In *cagC*, those codons with the highest likelihood of diversifying selection (amino acids 21 to 42; Table S5) overlap with codons forming surface-exposed and highly strain-specific epitopes in the N-terminus of mature CagC [38]. The *virB2* (HP0546) and *virB5* (HP0539) orthologs of the *cag*PAI show signatures of diversifying selection in the present study; they encode surface-exposed pilin and pilus tip structural components of the Cag apparatus [48] and their sequence homology with functionally related VirB2 and VirB5 proteins from other bacteria is so low that they had to be identified by non-sequence-based approaches [37], [38]. We also find that 9 other *cag*PAI genes are under diversifying selection but their function is largely unclear. These include HP0520, HP0522 (part of the Cag outer membrane subcomplex [49]), HP0523 (*cagγ*; proposed to code for a *virB1* orthologous peptidyglycan hydrolase [34], [50]), HP0528 (*virB9*), HP0534, HP0535, HP0536, HP0538 (encodes a membrane protein [50], [51]), and HP0540 [52]. Of these, HP0535 exhibits extensive non-synonymous variation and a clear bipartite Eastern-Western subdivision, similar to *cagA*. This gene is not involved in IL-8 induction or CagA translocation and is not predicted to possess a signal peptide. It may be a non-canonical secreted protein (score of 0.48 by SecretomeP). Based on the signs of selection and high diversity, we hypothesize that the HP0535-encoded protein interacts closely with CagA or is a novel effector protein that is translocated into host cells by the Cag t4ss. Of the other genes under diversifying selection whose function is unknown, HP0520 might be a non-canonical secreted protein because its SecretomeP score was also high (0.92).

In contrast to the genes just described, genes encoding *cag*PAI proteins that are not thought to be exposed on the bacterial surface [3] should be subject to purifying selection. In agreement with this expectation, other *cag*PAI genes including *virD4* (HP0524) and *virB11* (HP0525) orthologs [36], [50], displayed lower non-synonymous diversity and fewer codons under positive selection (Figure 6; Table S5).

In conclusion, the present work reports a genetic and functional approach within a global population genetic perspective to study diversity in a complex secretion system. This comprehensive library of data allowed the identification of genes with a high probability of having undergone diversifying selection. *cag*PAI genetic diversity is accompanied by modulations in functionality, but rarely by complete loss of function. Functional modulation of the t4ss appears to be an important feature *in vivo* and is predicted to rely not only on protein diversification but also on strain-dependent transcript level diversity in the *cag*PAI. These data will be a resource for future research on the biological roles and variable host interactions of individual *cag*PAI proteins. It will also foster research on the phylogeographic variability and evolution of determinants of host interaction in other microbes. The diversity in this dataset will also be useful to evaluating predictions by recent evolutionary models based on the structure of proteins, such as neutral networks of protein folds [53], [54]), which might be able to distinguish selection processes that favor structural *versus* functional conservation.

## Materials and Methods

### Bacterial isolates, sequencing, and RT–PCR

Bacterial isolates and sequences of seven housekeeping gene fragments (*atpA*, *efp*, *mutY*, *ppa*, *trpC*, *ureI*, *yphC*) have been described previously [13], [16], [55]. Strains were checked for the presence of the *cag*PAI by PCR, amplifying the 5′ (Primers O2872 + O2902) and 3′ (O2899 + O3326) flanking regions, or for absence (empty site) (primers O2872 + O3326). Primer sequences are provided in Table S1. Strains were chosen to represent all currently defined *H. pylori* populations possessing the *cag*PAI (Figure 1, Figure 2). The complete *cag*PAI was amplified for sequencing as two overlapping long range PCR products of ∼20 kb each with primers O2903 + O3048 and O3047 + O2904 (Table S1), respectively in 50 µl reactions with the EXL long range polymerase kit (Stratagene) using the following conditions: bacterial DNA 20 ng, Primers 20 µM each, 6 µl of 2mM dNTPs, 5 µl Buffer 1, 1 µl stabilizing solution, 1 µl EXL Polymerase, H_2_O to 50 µl. An initial denaturation for 1 min at 94°C was followed by 30 cycles of 45 sec at 94°C, 1 min at 65°C and 17 min 30 sec at 68°C. Long range PCR fragments were subjected to shotgun cloning. DNA fragments ranging from 0.8 to 1.2 kb were end repaired and cloned into the pGEM T-Easy vector (Promega), inserts were sequenced to 10-fold coverage by MWG Biotech. Alternatively, the *cag*PAIs were amplified as overlapping PCR products of ∼5 kb each with additional primers listed in Table S1 (primer combinations available on request) and sequenced with an extended set of primers (Table S1) by gene walking. The *cag*PAI sequence of strain PNGhigh85 was obtained by shotgun 454 sequencing of the whole genome (unpublished). Sequences were assembled with Gap4 (Staden Package, GCG Wisconsin). The individual cagPAI sequences have been submitted to the EMBL Nucleotide Sequence Database (accession numbers FR666825 - FR666857). Details for RNA preparation and RT-PCR are given in Text S1. RT-PCR primers and cycling conditions for transcript analyses of the *cag*PAIs are listed in Table S2.

### Multiple sequence alignment, sequence analysis, annotation, and phylogenetic analyses

CDSs were annotated in ACT and in KODON (Applied Maths BVBA, Sint-Martens-Latem, Belgium), automatic multiple sequence alignment of individual *cag*PAI genes was performed in BIONUMERICS (Applied Maths BVBA, Sint-Martens-Latem, Belgium) and corrected manually after visual inspection, where necessary. Sequence comparison and graphical output of multiple complete *cag*PAI sequences was performed in KODON. We only included one of eleven *cag*PAI sequences (F32) available from Japanese strains [19] because information is lacking on the phylogeographic population assignment of the remaining 10 strains. Pairwise genetic distances, phylogenetic trees and *F*_ST_ were calculated in MEGA3 [56] and in Arlequin [57], respectively. Pairwise geographic distances and distance from North East Africa (Addis Ababa, Ethiopia), as well as confidence intervals were calculated as previously described [16]. For analyses of increasing diversity with geographic distance from East Africa, the dataset was stripped of recent migrants [16] which resulted in the use of 33 out of the 37 *cag*PAI sequences. Pseudogenes were excluded from the dataset in all phylogenetic analyses.

### Evolutionary analyses

*K*s/*K*a ratios were determined in DnaSP4.0 [58] and SWAAP, including a sliding window analysis. The number and location of potential codons under selection (ω) in each *cag*PAI gene were determined using the program CODEML in PAML 3.15 [59], implementing a sliding windows graphic representation. This software calculates the ratio of maximum likelihood of different evolutionary algorithms (models) for each codon (site) of a coding sequence to be under positive selection (ω>1), followed by Naive Empirical Bayes (NEB) and Bayes Empirical Bayes (BEB) analyses of posterior probabilities. Sites with a posterior probability P>0.95 by the CODEML codon substitution models M3 (discrete) or M8 (beta and ω) of ω>1 were considered as being under positive or diversifying selection. The likelihood of codons under diversifying selection in the presence of recombination was further analyzed using OmegaMap (V 0.5; [32]). This software uses a Bayesian modeling algorithm to calculate the probability of codons to evolve under diversifying selection (ω>1) in the presence of recombination (ρ). By explicitly modeling recombination, this method has a low rate to detect false positives. The settings used in the program were: norders  = 100, thinning  = 100, rhoprior  =  inverse, omegaprior  =  inverse, block length  = 3 and 100,000 or 250,000 iterations. 5,000 iterations were deduced after each calculation as the burn-in phase. The model type used for both ω and ρ was “variable”. Three repetitions of the calculations with different settings were initially performed for control genes of defined structural properties and where some information is available about their function (e.g. HP0546), to exclude high variations in the calculations due to inadequate settings. Pseudogenes were excluded from the dataset.

### Housekeeping genes and population structure

Fragments of the housekeeping genes *atpA, efp, mutY, ppa, trpC, ureI*, and *yphC* were amplified and both strands were sequenced from independent PCR products as described [55]. Alternatively, comparable sequences were extracted from the published genomes (26695, HPAG1, J99). These sequences were assigned to populations and subpopulations by STRUCTURE [14].

### Functional assays of the *cag*PAI t4ss

IL-8 induction assay using the human gastric epithelial carcinoma cell line AGS (isolated from adenocarcinoma from a Caucasian patient) was performed for all strains of the sequencing project. Strain 26695A [60] was used as a reference. Cells were cultured in RPMI 1640 medium (buffered with 25 mM HEPES, supplemented with 10% heat-inactivated fetal bovine serum (medium and serum: Biochrom, Berlin, Germany). Details for bacterial culture conditions are given in Text S1. Cell infection experiments for IL-8 secretion measurement were performed on subconfluent cell layers (70%–90% confluence) in 24-well tissue culture plates. Cells were washed three times and preincubated in fresh medium with serum for 30 min prior to infection. By the addition of exponentially growing bacteria that were resuspended in cell culture medium (RPMI 1640, 25 mM HEPES, 10% heat-inactivated serum), the infection was started (MOI of 50). To synchronize the infection, the incubation plates were centrifuged at 500 x g, 20°C, for 3 min. The coincubation was carried out for 20 h. Non-infected cells (mock coincubated) were used as negative control. Supernatants were harvested, cleared of cell debris by centifugation, immediately frozen and stored at −20°C until use. Release of IL-8 into the cell supernatants was quantified by using BD OptEIA IL-8 enzyme-linked immunosorbent assay kit (BD Pharmingen; San Diego, USA) according to the company's instructions, using appropriate dilutions. The assays were performed in triplicate and the means and standard deviations of at least six independent coincubations were calculated. Adherence of the strains was tested in a high throughput assay, but no correlation was found between adherence and the IL-8 induction (data not shown).

To study CagA translocation, AGS cells were cultured in six-well plates and infected with *H. pylori* at a multiplicity of infection (MOI) of 100. After 4 h of coincubaction, non-adherent bacteria were removed by washing twice with PBS-Dulbecco (pH 7.4; Biochrom, Berlin, Germany). Cells were harvested with a cell scraper and resuspended in 1 ml PBS (pH = 7.4; Biochrom, Berlin, Germany). After centrifugation (250 x g, 4°C, 5 min), cells were resuspended in 300 µl of modified RIPA buffer (20 mM Tris-HCl [pH 7.5], 150 mM NaCl, 1 mM EDTA, 1 mM EGTA, 1% Triton X-100, 2.5 mM sodium pyrophosphate, 1 mM β-glycerol phosphate, 1 mM sodium orthovanadate, 1 protease inhibitor tablet per 10 ml buffer (Complete, Roche, Mannheim, Germany), 1 mM PMSF). During lysis, cells were incubated on ice for 30 min. Lysates were cleared by centrifugation (10 min, 21,900 x g, 4°) and the pellets were carefully separated from the supernatants. The pellet fraction was resuspended in 100 µl RIPA buffer and the fractions were immediately frozen at −80°C. To determine the amount of protein, a BCA protein assay was performed using the BCA Protein Assay kit (Pierce, Rockford, IL, USA) according to the manufacturer's instructions.

### Western blot analysis of CagA translocation

Equal amounts of cleared cell lysates (see above; corresponding to 10 µg of protein) of infected cells were resuspended in 5 x SDS loading buffer (0.31M Tris-HCl, pH6.8, 37.5% glycerol, 10% SDS, 0.05% bromophenol blue, 20% β-mercaptoethanol) and boiled for 10 min. For determination of molecular mass, BenchMark pre-stained Protein Ladder (Invitrogen, Karlsruhe, Germany) was used. Samples were separated on 10.4% denaturing SDS-polyacrylamide gels and transferred to nitrocellulose membranes (Protran BA 85, Whatman, Dassel, Germany) by semi-dry blotting. Membranes were blocked with 5% non-fat dried milk in TBS-T (20 mM Tris-HCl, 13.7 mM NaCl, 0.1% Tween 20, pH 7.4) for 1 h and subsequently incubated with specific primary antibody. Anti-CagA-antibody (Rabbit anti-*H. pylori* Cag antigen IgG fraction [polyclonal], Austral Biologicals, San Ramon, USA) was used at a dilution of 1/1,000 for the detection of CagA protein. To detect phosphorylated CagA, PY99-antibody (Santa Cruz Biotechnology, Heidelberg, Germany) was used (dilution 1/250). Goat-anti-Rabbit-HRP antibody (dilution 1/10,000, Jackson Immunoresearch Laboratories, Suffolk, Great Britain) or Goat-anti-mouse-HRP-antibody (dilution 1/5,000, Dianova, Hamburg, Germany) were used as secondary antibodies. Signal detection was performed with Enhanced SuperSignal West chemiluminescence substrate (Pierce, Rockford, IL, USA), and detection was on X-ray film (Hyperfilm, Amersham Biosciences, Buckinghamshire, UK).

## Supporting Information

Figure S1 Distribution of IS and mini IS elements and repetitive sequences in diverse *cag*PAIs. Repetitive sequences and sites where insertion (IS) elements and mini IS elements have integrated are indicated by symbols. Green: *cag*PAI insertion site containing repetitive sequence; red rectangles: mini IS*606* insertions; blue triangles: mini IS605 insertion sites. Mini-IS*607* and mini IS*608* elements were not identified. a,b,c,d,e: different genetic variants of IS*606* insertion elements.
(0.14 MB PDF)

Table S1 List of primers.
(0.04 MB XLS)

Table S2 Primer list for transcript analyses of *cag*PAI genes.
(0.02 MB XLS)

Table S3 Transcript table for selected *cag* genes with a role in cag t4ss function (IL-8 induction) and for *cag*A.
(0.02 MB XLS)

Table S4 List of all identical alleles in single *cag* genes of the 38 analyzed *cag*PAIs.
(0.03 MB DOC)

Table S5 Congruence between PAML (CODEML model M8) and OmegaMap analyses for probabilities of diversifying selection of sites in *H. pylori cag*PAI genes.
(0.04 MB XLS)

Text S1 Supplementary Materials and Methods.
(0.02 MB DOC)
