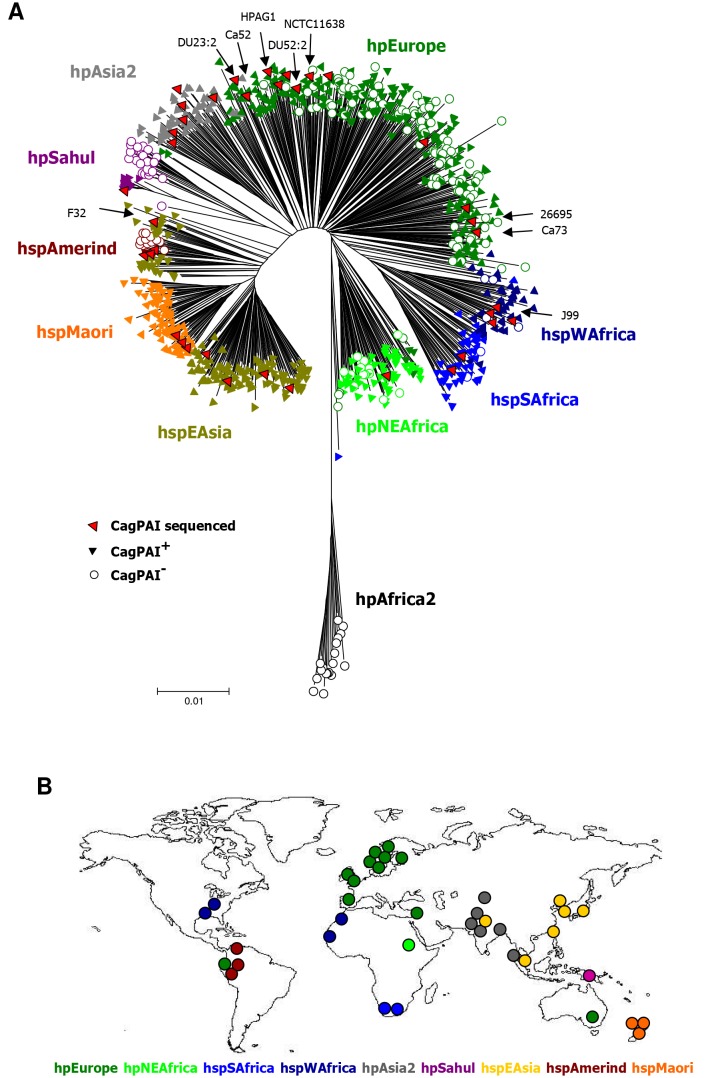# Correction: A Global Overview of the Genetic and Functional Diversity in the *Helicobacter pylori cag* Pathogenicity Island

**DOI:** 10.1371/annotation/3597bc55-d182-4838-b360-f739efddcb4e

**Published:** 2014-01-03

**Authors:** Patrick Olbermann, Christine Josenhans, Yoshan Moodley, Markus Uhr, Christiana Stamer, Marc Vauterin, Sebastian Suerbaum, Mark Achtman, Bodo Linz

In Table 1 deletion 11 was mislabeled as deletion 3. Deletion 11 was identified in strain CC42C, rather than deletion 3 in strain PNGhigh85. Deletion 11 truncates the genes cagE, cagD and cagC, thus abolishing the function of the T4SS. In contrast, deletion 3 in PNGhigh85 is at (J99) nucleotide positions 798 to 1418, similar to the end points of deletions 1 and 2, and like those deletions does not affect IL-8 induction. This error was brought to our attention by Abbas Yadegar, who has posted the same information in a comment.

Another error was introduced into Figure 1A. In the Neighbor joining tree, some of the Australian hpSahul strains are cagPAI positive and some are cagPAI negative ("upper" clade in hpSahul). Re-evaluation of the results indicated that all analyzed Australian hpSahul strains are cagPAI negative (open circles). In contrast, all analyzed hpSahul strains from Papua New Guinea ("lower" clade in hpSahul) are cagPAI positive (filled triangle), as was previously stated in the text. The corrected Figure 1A can be seen here:

**Figure:**