# Supplementary figures and images for: A Global Overview of the Genetic and Functional Diversity in the Helicobacter pylori cag Pathogenicity Island
Source: PLoS Genet. 2010 Aug 19;6(8):e1001069. doi: 10.1371/journal.pgen.1001069 (PMC2924317; doi:10.1371/journal.pgen.1001069)

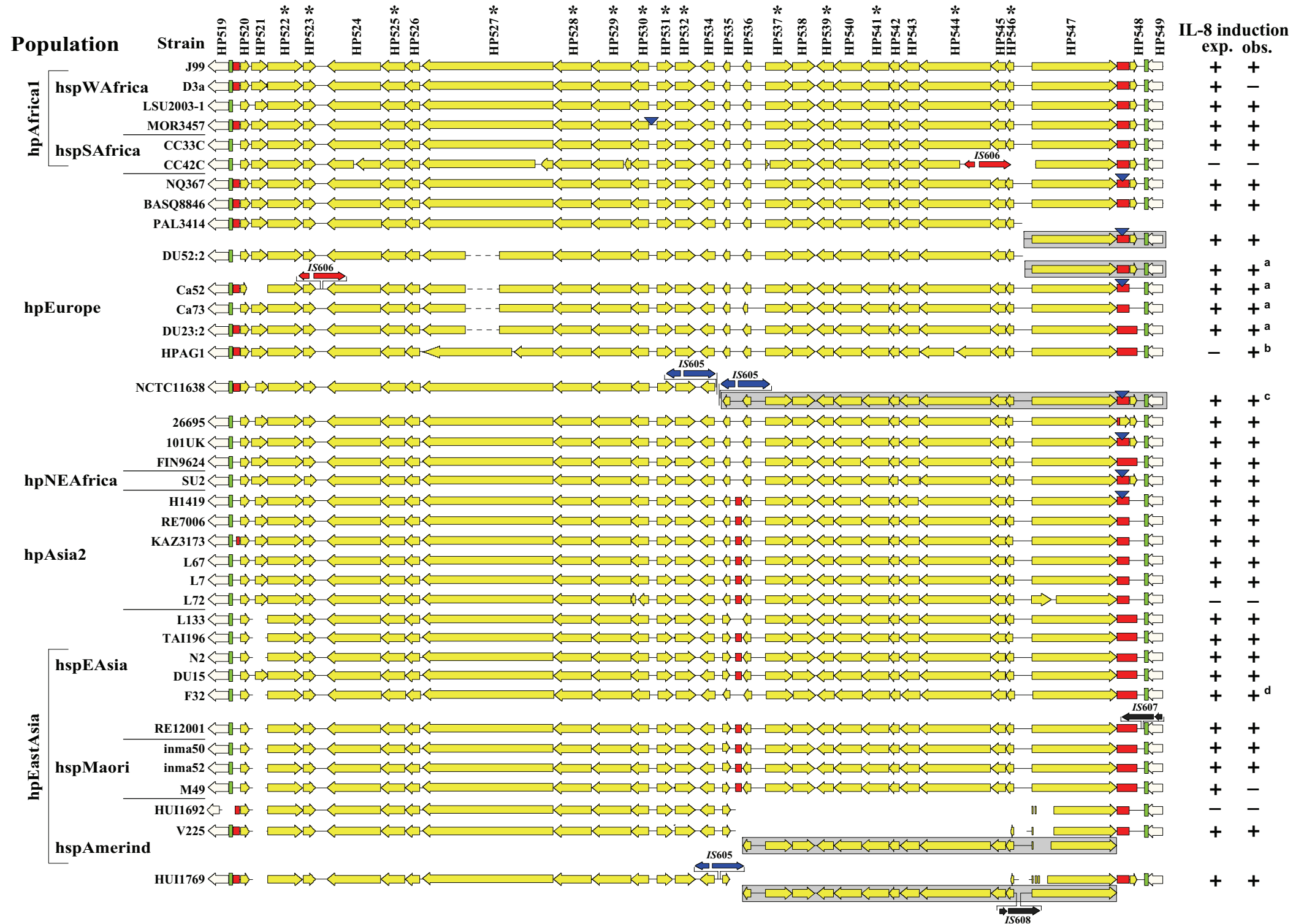

Supplement: Figure S1 — Distribution of IS and mini IS elements and repetitive sequences in diverse cagPAIs. Repetitive sequences and sites where insertion (IS) elements and mini IS elements have integrated are indicated by symbols. Green: cagPAI insertion site containing repetitive sequence; red rectangles: mini IS606 insertions; blue triangles: mini IS605 insertion sites. Mini-IS607 and mini IS608 elements were not identified. a,b,c,d,e: different genetic variants of IS606 insertion elements. (0.14 MB PDF) [file pgen.1001069.s001.pdf]
