# Supplementary material for: A Global Overview of the Genetic and Functional Diversity in the Helicobacter pylori cag Pathogenicity Island
Source: PLoS Genet. 2010 Aug 19;6(8):e1001069. doi: 10.1371/journal.pgen.1001069 (PMC2924317; doi:10.1371/journal.pgen.1001069)
Supplement: Table S4 — List of all identical alleles in single cag genes of the 38 analyzed cagPAIs. (0.03 MB DOC) [file pgen.1001069.s005.doc]

| Gene number | Number of identical alleles in 38 analyzed *cag*PAI sequences | Strain names | Population assignment |
| --- | --- | --- | --- |
| HP0520 | 2 | Inma52, M49 | hpEastAsia, hpEastAsia |
| HP0523 | 2 | Inma52, Inma50 | hpEastAsia, hpEastAsia |
| HP0531 | 2 | Inma52, M49 | hpEastAsia, hpEastAsia |
| HP0533 | 2 | HUI1692, HUI1769 | hpEastAsia, hpEastAsia |
| HP0535 | 2 | 11638, HPAG1 | HPEurope, HPEurope |
| HP0538 | 2 | Inma50, Inma52 | hpEastAsia, hpEastAsia |

**Suppl. Table S4:** list of all identical alleles in single *cag* genes of the 38 analyzed *cag*PAIs. Note that identical alleles were predominantly found in the hpEastAsia population, potentially indicating a selective sweep.
