# Supplementary material for: A Global Overview of the Genetic and Functional Diversity in the Helicobacter pylori cag Pathogenicity Island
Source: PLoS Genet. 2010 Aug 19;6(8):e1001069. doi: 10.1371/journal.pgen.1001069 (PMC2924317; doi:10.1371/journal.pgen.1001069)
Supplement: Text S1 — Supplementary Materials and Methods. (0.02 MB DOC) [file pgen.1001069.s007.doc]

**Supplementary Materials & Methods to Olbermann et al.**

**Bacterial culture conditions.** Bacteria were grown under microaerophilic conditions (5% O2, 10% CO2, 85% N2) in a Scholzen Anaerobic Incubator (Scholzen Microbiology Systems, Wittenbach, Switzerland) on blood agar plates (Columbia agar base II; Oxoid, Wesel, Germany) supplemented with 10% (v/v) defibrinated horse blood and containing the following antibiotics: vancomycin (10 mg/liter), polymyxin B (2,500 U/liter), trimethoprim (5 mg/liter) and amphotericin B (4 mg/liter). For infection experiments, *H. pylori* strains were preincubated for 24 h at 37°C under microaerobic conditions on plates. Bacteria used for gene expression analysis were cultivated in liquid culture in brain-heart infusion (BHI) broth (Oxoid, Wesel, Germany) supplemented with 10% heat-inactivated horse serum (Biochrom, Berlin, Germany), 5% (w/v) yeast extract and the required antibiotics (see above). Liquid cultures used for RNA preparation were incubated at 37°C in anaerobic jars under microaerophilic conditions generated by Anaerocult C gas generating sachets (Merck, Darmstadt, Germany) in a shaking incubator (200 rpm) until the cultures reached an O.D.600nm of approximately 1.0 (mid exponential growth) or 2.0 (late exponential growth).

**Transcript analysis of selected *cag*PAI genes with a role in t4ss function - RNA preparation and RT-PCR.** Total RNA was prepared from bacteria exponentially grown in liquid culture (OD_600_ = 1) and disrupted in Lysing Matrix B tubes (MP Biochemicals, Heidelberg, Germany) in a FastPrep® FP120 instrument (MP Biochemicals, Heidelberg, Germany) for 45 s, at 6.5 m/s. Afterwards, the Qiagen RNeasy spin prep kit was used for further purification of whole RNA with slight modifications (Josenhans et al., 2002). Semiquantitative RT-PCRs were performed on 2 µg of DNase I-treated RNA samples. cDNA synthesis was performed for 2 h at 42°C using a random hexamer primer mix (300 ng) and Superscript III TM reverse transcriptase (200 U; both Invitrogen, Karlsruhe Germany). To inactivate the enzyme, cDNA samples were incubated for 10 min at 70°C and adjusted with ddH2O to a final volume of 50 µl. PCRs were performed on 1.0 µl cDNA sample with primers specific for the corresponding genes (primers and amplification conditions: Supplementary Table S2). Amplification was not possible for all genes in all strains with one primer pair, therefore several different primer pairs were developed to amplify all tested genes from all strains. Functionality of primers for detection of specific *cag*PAI gene transcripts were first controlled with genomic DNA of the investigated strains. This was achieved for all strains with one or both of the primer pairs used. The appropriate cycle number to achieve a visible transcript signal in agarose gels after RT-PCR was tested for each gene separately (see Supplementary Table S2). All RT-PCRs were performed with strain 88-3887 (26695A) cDNA as positive and water as negative controls. Each cDNA-sample concentration was tested for integrity by amplification of 16S-ribosomal cDNA and comparison of signal intensity.
